# The efficacy and safety of intravenous administration of tranexamic acid in patients undergoing cardiac surgery: Evidence from a single cardiovascular center

**DOI:** 10.1097/MD.0000000000033819

**Published:** 2023-05-17

**Authors:** Pei-Shuang Lin, Yun-Tai Yao, Li-Juan Tian, Juan-Juan Jiang, Yang Zhang, Li-Xian He, Yi-Ping Yu, Jie Ma

**Affiliations:** a Department of Anesthesiology, Fuwai Hospital, National Center for Cardiovascular Diseases, Peking Union Medical College and Chinese Academy of Medical Sciences, Beijing, China; b Department of Cardiovascular surgery, Fujian Medical University Affiliated First Quanzhou Hospital, Fujian, China; c Key Laboratory of Clinical Research for Cardiovascular Medications, Fuwai Hospital, National Center for Cardiovascular Diseases, Peking Union Medical College and Chinese Academy of Medical Sciences, Beijing, China; d Department of Laboratory Medicine, National Center for Cardiovascular Diseases, Peking Union Medical College and Chinese Academy of Medical Sciences, Beijing, China; e Department of Anesthesiology, Fuwai Yunnan Cardiovascular Hospital, Kunming, China; f Department of Pharmacy, Fuwai Hospital, National Center for Cardiovascular Diseases, Peking Union Medical College and Chinese Academy of Medical Sciences, Beijing, China.

**Keywords:** bleeding, cardiac surgery, meta-analysis, tranexamic acid, transfusion

## Abstract

The current study was performed to systemically review the efficacy and safety of tranexamic acid (TXA) in patients undergoing cardiac surgery at a single large-volume cardiovascular center.

**Methods:**

A computerized search of electronic databases was performed to identify all relevant studies using search terms till December 31^st^, 2021. The primary outcomes were postoperative blood loss and the composite incidence of mortality and morbidities during hospitalization. Secondary outcomes included postoperative massive bleeding and transfusion, postoperative recovery profiles, coagulation functions, inflammatory variables, and biomarkers of vital organ injury.

**Results:**

Database search yielded 23 qualified studies including 27,729 patients in total. Among them, 14,136 were allocated into TXA group and 13,593 into Control group. The current study indicated that intravenous TXA significantly reduced total volume of postoperative bleeding in both adult and pediatric patients, and that medium- and high-dose TXA were more effective than low-dose TXA in adult patients (*P* < .05). The current study also demonstrated that intravenous TXA, as compared to Control, remarkably reduced postoperative transfusion incidences and volume of red blood cell and fresh frozen plasma, and reduced postoperative transfusion incidence of platelet concentrates (PC) (*P* < .05) without obvious dose-effects (*P* > .05), but TXA did not reduce PC transfusion volume postoperatively in adult patients (*P* > .05). For pediatrics, TXA did not significantly reduce postoperative transfusion incidence and volume of allogenic red blood cell, fresh frozen plasma and PC (*P* > .05). Additionally, the current study demonstrated that intravenous TXA did not influence the composite incidence of postoperative mortality and morbidities in either adults or pediatrics during hospitalization (*P* > .05), and that there was no obvious dose-effect of TXA in adult patients (*P* > .05).

**Conclusions:**

This current study suggested that intravenous TXA significantly reduced total volume of postoperative bleeding in both adult and pediatric patients undergoing cardiac surgery at the single cardiovascular center without increasing the composite incidence of mortality and morbidities.

## 1. Introduction

Systemic heparinization, hemodilution and hypothermia during cardiac surgery significantly influence coagulation and fibrinolysis systems,^[1]^ which leads to excessive bleeding and increasing need for allogeneic transfusion. Blood loss and transfusion requirement are important quality control indicators of cardiovascular surgery and anesthesia.^[2]^ Tranexamic acid (TXA), a synthetic lysine analogue, binds to plasminogen, prevents tissue-type plasminogen activator mediated release of active plasmin and therefore inhibit fibrinolysis.^[3]^ As an important pharmacological adjunct recommended by the World Health Organization,^[4]^ TXA is currently the most widely used antifibrinolytic agent and the mainstay hemostatic agent to prevent or decrease perioperative bleeding at Fuwai Hospital. Though numerous studies and extensive literature reviews have proved the efficacy of TXA in reducing bleeding and allogeneic blood transfusion requirements in patients receiving various surgical procedures, many controversies persist, for instance, the optimal administration regimens of this drug and safety concerns.^[5]^ Particularly in cardiovascular surgical patients with high-risk for bleeding, the dose-effects and safety profiles of TXA have been broadly investigated yet still need further scrutiny.^[6–10]^ Thus, to provide further evidence, we conducted a systemic review and meta-analysis to evaluate the efficacy and safety of intravenous TXA in patients undergoing cardiac surgery at the single large-volume cardiovascular center.

## 2. Materials and methods

### 2.1. Ethical approval

Ethical approval was not necessary because this study was a systemic review of previously published literatures.

### 2.2. Search strategy

The protocol of the current meta-analysis was published in PROSPERO with the registration number of CRD42022322273. The Research and Information Sharing Platform, an internal database recording all institutional publications ever since its establishment, was searched to identify all clinical studies concerning intravenous TXA in cardiac surgical patients till December 31^st^, 2021. Furthermore, MEDLINE, Cochrane Library, EMBASE, and OVID were searched, using search words as follows: [(tranexamic acid) OR (antifibrinoli*)] *AND* [(*cardiopulmonary bypass) OR (heart) OR (cardiac surgery) OR (coronary artery bypass surgery) OR (valve surgery) OR (aortic surgery*)] *AND (Fuwai*) without language restriction till December 31^st^, 2021. We also searched Chinese Bio Medical Literature & Retrieval System (from 1978 to December 31^st^, 2021). Additionally, we used the bibliography of retrieved articles to further identify qualified studies. All database search was updated on January 31^st^, 2022.

### 2.3. Inclusion and exclusion criteria

We included all published clinical studies reporting intravenous administration of TXA as compared to Control (placebo/blank) or other doses of TXA in patients undergoing cardiovascular surgery at Fuwai Hospital. We excluded studies published as narrative review, meta-analysis, guidelines, expert opinions, case report, protocol or abstract, animal or cell studies, and studies lacking outcome data. Two authors (YTY and PSL) independently assessed all identified studies for eligibility. Disagreement was resolved by consensus or the opinion of a third reviewer.

### 2.4. Study quality assessment

Two authors (YTY and PSL) independently assessed the risk of bias of each study. Cochrane Handbook for Systematic Reviews of Interventions^[11]^ was used for randomized control trials and the Newcastle Ottawa Scale ^[12]^ for nonrandomized studies to assess the methodological quality of included studies.

### 2.5. Outcomes, TXA dose range and data abstraction

Total volume of postoperative blood loss and the composite incidence of mortality and morbidities during hospitalization were defined as primary efficacy outcome and safety outcome, respectively. Secondary outcomes of interest included other bleeding and transfusion variables [e.g., postoperative blood loss within 8 hours and 24 hours, the incidences of postoperative massive bleeding, reoperation for bleeding, and transfusion of allogenic red blood cell (RBC), fresh frozen plasma (FFP), and platelet concentrates (PC)], postoperative recovery profiles [e.g., mechanical ventilation duration, length of stay (LOS) in the intensive care unit and hospital, composite incidence of mortality and morbidities during long-term follow-ups], coagulation functions [e.g., intraoperative heparin and protamine doses, postoperative coagulation tests, platelet (Plt) count and function], inflammatory variables [e.g., white blood cell (WBC) count, interleukins (ILs)], and biomarkers of vital organ injury[e.g., cardiac troponin (cTn), creatinine].

To evaluate its dose-effects on the outcomes, the dose ranges of TXA were defined as described: “Low-Dose” = [Bolus dose of (0–10) mg/kg and maintenance dose of (0–10) mg/kg/hours], “Medium-Dose”=[Bolus dose of (10–20) mg/kg and maintenance dose of(10–15) mg/kg/hours], and “High-Dose”=[Bolus dose of (20–30)mg/kg and maintenance dose of (15–20) mg/kg/hours].^[6,13,14]^

Two authors (YTY and PSL) independently abstracted the following data; First author and publication year; Study design; Cardiac surgical procedures; Total number of patients, and number of patients in TXA and Control groups; Data regarding outcomes of interest. Disagreement was resolved during the process of abstraction.

### 2.6. Statistical analysis

Meta-analysis was performed to evaluate the efficacy and safety of intravenous TXA among finally included studies. All data were analyzed by utilizing Rev Man 5.4 (Cochrane Collaboration, Oxford, UK). Continuous data were presented as weighted mean difference and 95% confidence interval, while dichotomous data as pooled odds ratio and 95% confidence interval. Statistical heterogeneity was evaluated for each outcome and randomized-effects or fixed-effects model was used in the presence or absence of significant heterogeneity (*I*^2^ > 50%). Sensitivity analyses were conducted by examining the influence of statistical model on estimated treatment effects. In addition to that, we also performed sensitivity analysis to evaluate the influence of individual study on the overall effects. Statistical significance was defined as *P* < .05.

## 3. Results

### 3.1. Search results

**Figure 1.**
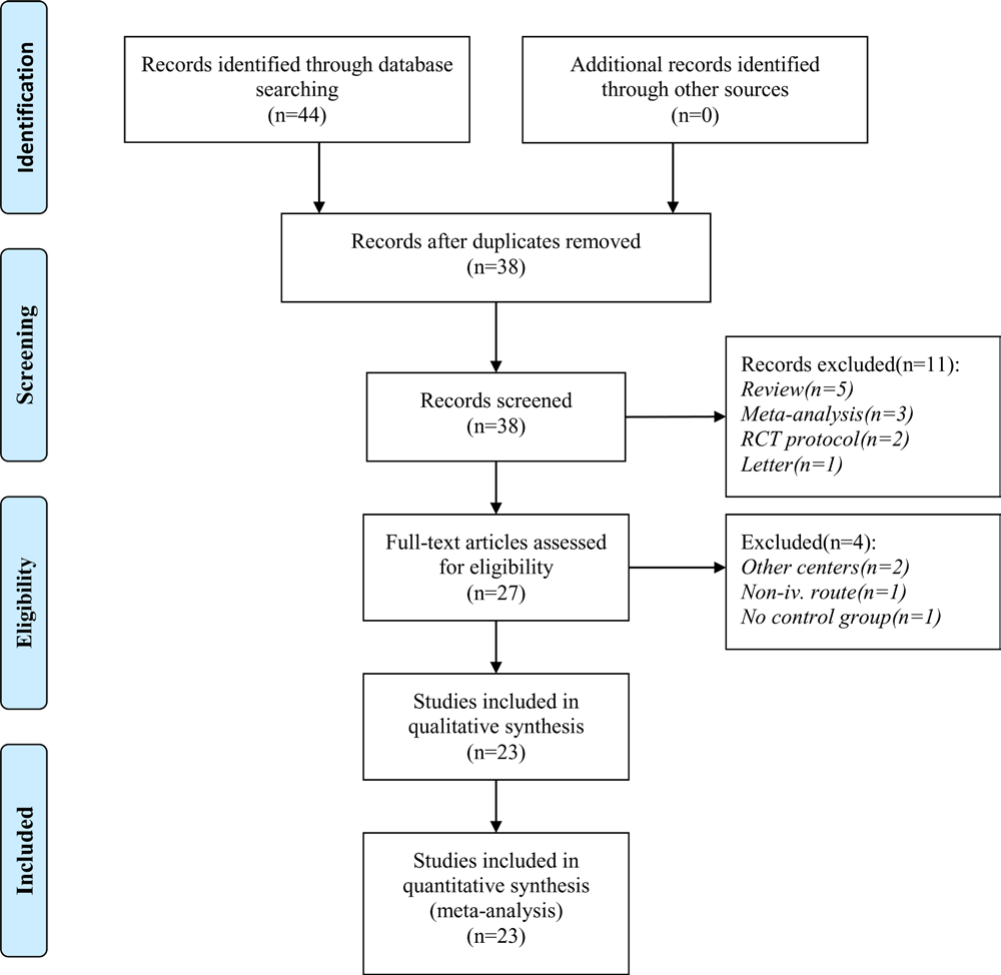
Flow chart.

As depicted in the flow chart (Fig. 1), research and information sharing platform database search identified 44 articles for complete evaluation. Finally, 23 eligible studies^[15–37]^ were included for analysis. Characteristics of these articles were presented in Table 1.

**Table 1:** Characteristics of includes studies.

References	Design	Surgical procedures (*Patients*)	Antiplatelet therapy	N	Group TXA	Group control
Zhang P 2020^[15]^	RCT(DB)	Primary, elective on-pump CABG, VS, CHD-R (*Adults*)	NR	284	TXA [Low-dose] (n = 143) 15 mg/kg *iv.* after AI, 15 mg/kg *iv.* after protamine	Saline (n = 141)
Tian LJ 2020^[16]^	RCT(DB)	Primary, elective on-pump CABG, VR (*Adults*)	*Discont.* ≥7 d	139	①TXA [Low-dose] (n = 46): 10 mg/kg *iv*. after AI, followed by 10 mg/kg/h *iv*. till EOP ②TXA [Medium dose] (n = 46): 20 mg/kg *iv*. after AI, followed by 15 mg/kg/h *iv*. till EOP	Saline (n = 47)
Lv H 2020^[17]^	RCT(DB)	Primary, elective on-pump CABG, VS, (*Adults*)	*Discont.* ≥7 d	40	TXA [Medium dose] (n = 20): 20 mg/kg *iv.* after AI, followed by 15 mg/kg/h till EOP	Saline (n = 20)
Lv H 2019a^[18]^	RCT(DB)	Primary, elective on-pump VR (*Adults*)	*Discont.*>7 d	126	①TXA[Low-dose] (n = 31): 10 mg/kg *iv*. after AI, followed by 10 mg/kg/h *iv*. till EOP ②TXA [Medium dose] (n = 31): 20 mg/kg *iv*. after AI, followed by 15 mg/kg/h *iv*. till EOP ③TXA [High-dose] (n = 33): 30 mg/kg *iv*. after AI, followed by 20 mg/kg/h *iv*. till EOP	Saline (n = 31)
Lv H 2019b^[19]^	RCT(DB)	Primary, elective on-pump CABG, VS, (*Adults*)	*Discont.* ≥7 d	101	TXA [Low-dose] (n = 48): 10 mg/kg *iv.* after AI, followed by 10 mg/kg/h till EOP	Saline (n = 53)
Lv H 2019c^[20]^	RCT(DB)	Primary, elective on-pump CABG, VS, (*Adults*)	*Discont.* ≥7 d	101	TXA [Low-dose] (n = 51): 10 mg/kg *iv.* after AI, followed by 10 mg/kg/h till EOP	Saline (n = 50)
Zhang Y 2018d^[21]^	RCT(TB)	Primary, elective on-pump CABG (*Adults*)	*Discont .*≥7 d	202	TXA[Low-dose](n = 100): 10 mg/kg *iv.* after AI, followed by 10 mg/kg/h till EOP	Saline(n = 102)
Zhou Y 2018^[22]^	RCT(DB)	Primary, elective on-pump CABG, VS (*Adults*)	*Discont.*>7 d	150	①TXA [Low-dose] (n = 50): 10 mg/kg *iv*. after AI, followed by 10 mg/kg/h *iv*. till EOP ②TXA [Medium dose](n = 50): 20 mg/kg *iv*. after AI, followed by 15 mg/kg/h *iv*. till EOP ③TXA [High-dose] (n = 50): 30 mg/kg *iv*. after AI, followed by 20 mg/kg/h *iv*. till EOP	None
Wang J 2017^[23]^	RCT(DB)	Primary, elective on-pump CABG (*Adults*)	No exposure, *Discont.* ≥7 d	211	TXA [Low-dose] (n = 105): 10 mg/kg *iv.* after AI, followed by 10 mg/kg/h till EOP	Saline (n = 106)
Shi J 2013a^[24]^	RCT(DB)	Primary, elective on-pump CABG (*Adults*)	DAPT *Discont*.<7 d	110	TXA [Low-dose] (n = 55): 10 mg/kg *iv.* after AI, followed by 10 mg/kg/h till EOP	Saline (n = 55)
Shi J 2013b^[25]^	RCT(DB)	Primary, on-pump CABG (*Adults*)	DAPT *Discont*. <7 d	117	TXA [Low-dose] (n = 58): 15 mg/kg iv. before SI, 15 mg/kg *iv*. after protamine	Saline (n = 59)
Shi J 2013c^[26]^	RCT(SB)	On-pump CABG (*Adults*)	CLO *Discont*. <7/≥7 d	120	TXA [Low-dose] (n = 60): 10 mg/kg *iv.* after AI, followed by 10 mg/kg/h till EOP *CLO exposure* (n = 60)	Saline (n = 60) *Clopidogrel exposure* (n = 60)
Shi J 2013d^[27]^	RCT(DB)	Primary, elective/emergency On-pump CABG (*Adults*)	①*CLO Discon. ≤7 d* ②*CLO Discon. >7 d* ③*No CLO exposure*	552	TXA [Low-dose] (n = 274): 10 mg/kg *iv.* after AI, followed by 10 mg/kg/h till EOP ①*CLO Discon. ≤7 d*(n = 63) ②*CLO Discon.>7 d* (n = 105) ③*No CLO exposure* (n = 106)	Saline (n = 278) ①*CLO Discon. ≤7 d* (n = 65) ②*CLO Discon. >7 d* (n = 106) ③*No CLO exposure* (n = 107)
Du YJ 2013^[28]^	RCT(DB)	Primary, elective valvular surgery (*Adults*)	No exposure	150	①TXA [Low-dose] (n = 49): 10 mg/kg *iv*. after AI, followed by 2 mg/kg/h *iv*. till EOP ②TXA [Medium dose] (n = 51): 15 mg/kg *iv*. after AI, followed by 8 mg/kg/h *iv*. till EOP ③TXA [High-dose] (n = 50): 30 mg/kg *iv*. after AI, followed by 16 mg/kg/h *iv*. till EOP	None
Wang GY 2012^[29]^	RCT(DB)	Primary, elective off-pump CABG (*Adults*)	*Discont*. ≥5 d	231	TXA [Low-dose] (n = 116) 1 g *iv.* before SI, then 400 mg/h till EOP	Saline (n = 115)
Wang GY 2011^[30]^	RCT(SB)	Primary, elective off-pump CABG (*Adults*)	*Discont*. ≥7 d	60	TXA [Low-dose] (n = 30) 1 g *iv.* after AI, then 400 mg/h till EOP	Saline (n = 30)
Li CY 2011^[31]^	RCT(SB)	Off-pump CABG (*Adults*)	NR	140	TXA [Low-dose] (n = 70): 2 mg/kg *iv.* after AI, followed by 28mg/kg *iv.* till EOP	Saline (n = 70)
Yue J 2005^[32]^	RCT(DB)	Primary, elective ASD-, VSD-R (*Pediatrics*)	NR	30	TXA [Low-dose] (n = 15): 10 mg/kg *iv*. before CPB, 10 mg/kg *iv*. during CPB	Saline (n = 15)
Wang ES 2021^[33]^	Retrospective (PSM)	Primary, on- and off-pump CABG (*Adults*)	NR	21,038	TXA (n = 10,519): ①TXA [Low, Medium dose] (n = 8645): TXA dose < 50 mg/kg *iv*. ②TXA [High-dose] (n = 8645): TXA dose ≥ 50 mg/kg iv.	BLK (n = 10,519): No antifibrinolytics
Liu Y 2021^[34]^	Retrospective	Primary, on-pump CS (*Adults*)	NR	237	①TXA [Low-dose] (n = 80): Bolus: (0-10) mg/kg *iv*., maintenance: (0-10) mg/kg/h *iv*. ②TXA [Medium dose] (n = 78): Bolus: (10-20) mg/kg iv., maintenance: (10-15) mg/kg/h iv. ③TXA [High-dose] (n = 79): Bolus: (20-30) mg/kg *iv*., maintenance: (15-20) mg/kg/h *iv*.	None
Zhang Y 2019^[35]^	Retrospective	Primary, ASD-, VSD-, TOF-R (*Pediatrics*)	NR	2026	TXA [Medium dose] (n = 970): 15 mg/kg/h *iv.* after AI till end of CPB	BLK (n = 1056): No antifibrinolytics
Zhang Y 2018a^[36]^	Retrospective	TOF-R (*Pediatrics*)	NR	328	TXA [High-dose] (n = 190): (50-80) mg/kg *iv.* after AI till end of CPB	BLK (n = 138): No antifibrinolytics
Zhang Y 2018b^[37]^	Retrospective	VSD-R (*Pediatrics*)	NR	1236	TXA [High-dose] (n = 588): (50-80) mg/kg *iv. pump* after AI	BLK (n = 648): No antifibrinolytics

AI = anesthesia induction, ASD = atrial septal defect, BLK = blank, CABG = coronary artery bypass, CHD = congenital heart diseases, CLO = Clopidogrel, CPB = cardiopulmonary bypass, CS = cardiac surgery, DAPT = dual antiplatelet therapy, DB = double-blinded, *Discon.* = discontinuation, EOP = end of procedure, *iv* = intravenously, N = sample size, NR = not reported, PSM = propensity score matching, R = repair, RCT = randomized controlled trial, SB = single-blinded, SI = skin incision, TB = triple-blinded, TOF = tetralogy of Fallot, TXA = tranexamic acid, VR = valve repair, VS = valve surgery, VSD = ventricular septal defect.

### 3.2. Included trials characteristics

As shown in Table 1, 7^[15,21,25,27,29,33,35]^ of the 23 literatures were written in English, and the other 16^[16–20,22–24,26,28,30–32,34,36,37]^ were in Chinese. Eighteen studies were randomized controlled trials (RCTs), and 5 were observational studies. Among the 23 studies, 10 included only adult patients undergoing coronary artery bypass graft (CABG) (6 on-pump,^[21,23–27]^ 3 off-pump^[29–31]^ and 1 mixed on-pump and off-pump^[33]^), 7^[16–20,22,28]^ included adult patients undergoing valve surgery with or without CABG, 2^[15,17]^ included adult patients undergoing mixed types of cardiac surgeries, 4^[32,35–37]^ included only pediatric patients undergoing correction for congenital heart diseases (2 non-cyanotic,^[32,37]^ 1 cyanotic^[36]^ and 1 mixed cyanotic and non-cyanotic^[35]^).

As shown in Table 1, 11 of the 23 studies^[21–28,33–35]^ enrolled only patients who never receive or discontinued antiplatelet therapy (aspirin, and/or clopidogrel) preoperatively, 2 studies^[24,25]^ investigated the effects of TXA in on-pump CABG patients who ingested dual antiplatelet therapy (aspirin and clopidogrel) within 7 days preoperatively, 2 studies^[26,27]^ examined the interaction of TXA and clopidogrel by stratifying on-pump CABG patients into 3 levels (clopidogrel ingestion ≤ 7 days, clopidogrel discontinuation > 7 days, and no exposure), the other studies^[15,31–37]^ did not provide antiplatelet information. These 23 eligible studies involved totally 27,729 patients, 14,136 of whom were allocated into TXA group and the other 13,593 into Control (placebo/blank) group. TXA dose regimes varied among the 23 studies. For example, 16 studies,^[15,16,18–21,23–27,29–33]^ 4 studies^[16–18,35]^ and 4 studies^[18,33,36,37]^ investigated the effects of low-, medium- or high-dose TXA as compared to Control, respectively. The studies by Lv et al,^[13]^ Zhou et al,^[22]^ Du et al^[28]^ and Liu et al^[34]^ measured all low-, medium- and high-doses of TXA, the study by Tian et al^[16]^ measured both low- and medium-doses of TXA.

### 3.3. Quality assessment

Risk of bias assessment of the 18 RCTs using the Cochrane Collaboration’s tool was shown in Figure S1, Supplemental Digital Content, and Figure S2, Supplemental Digital Content, https://links.lww.com/MD/J4. Most of the trials included had low risk of bias on random sequence generation, incomplete outcome data and selective reporting. Possible risk of bias mainly lied in allocation concealing and binding. The quality assessment of the 5 observational studies was shown in Table S1, Supplemental Digital Content, http://links.lww.com/MD/J5. Scores of these studies ranged from 7 to 9, indicating low risk of bias according to the Newcastle Ottawa Scale scoring system.

### 3.4. Effects on postoperative bleeding

**Figure 2.**
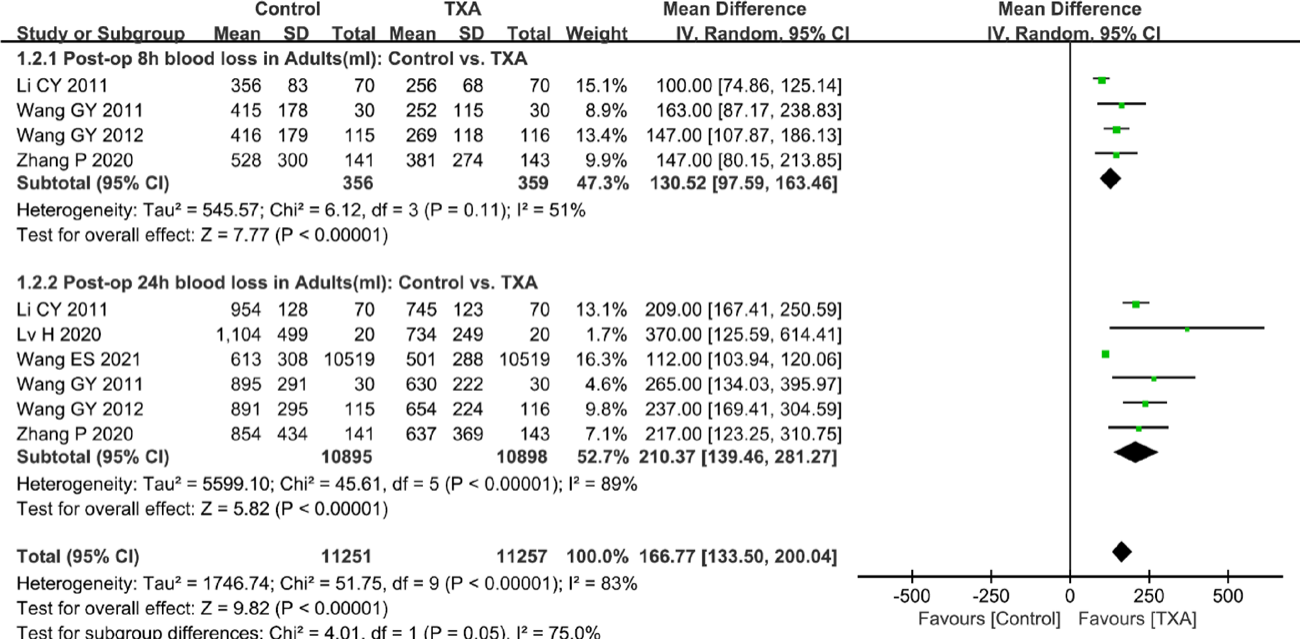
Forest plot of postoperative blood loss within 8- and 24-h in adults.

**Figure 3.**
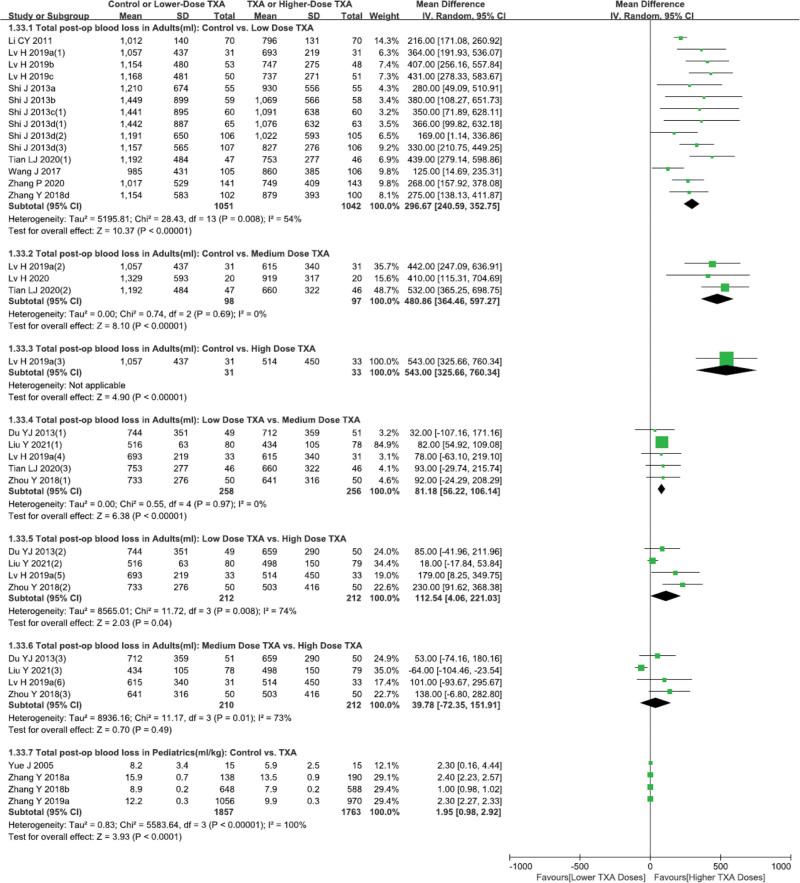
Forest plot of total postoperative blood loss in adults and pediatrics.

**Figure 4.**
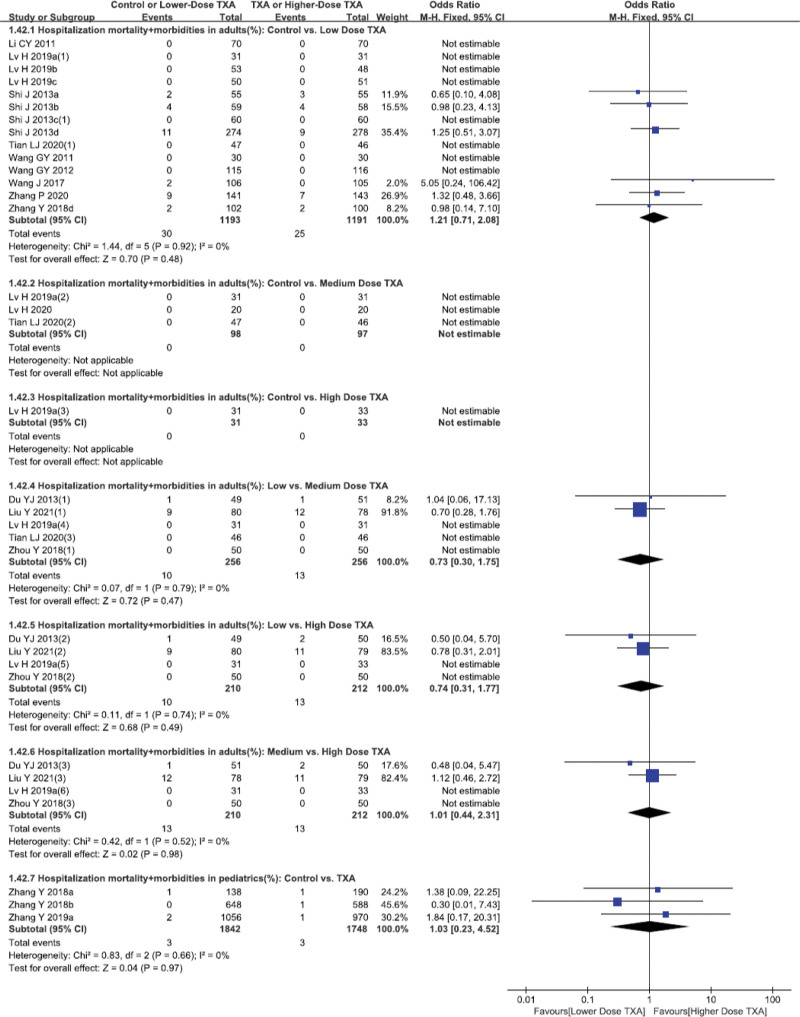
Forest plot of the composite incidence of mortality and morbidities in adults and pediatrics during hospitalization.

As shown in Figures 2 and 4 studies^[15,29–31]^ (715 patients) and 6 studies^[15,17,29–31,33]^ (21,793 patients) examined the effect of TXA versus Control in adults on reducing blood loss within 8 hours and 24 hours postoperatively. Meta-analysis indicated that TXA, as compared to Control, significantly reduced blood loss within 8 hours and 24 hours postoperatively. As shown in Figures 3 and 12 studies^[15,16,18–21,23–27,31]^ (2093 patients), 3 studies^[16–18]^ (197 patients) and 1 study^[23]^ (64 patients) examined the effect of low-, medium- and high-dose TXA versus Control in adults with respect to total volume of postoperative bleeding. Meta-analysis demonstrated that, all 3 doses of TXA significantly reduced total blood loss postoperatively as compared to Control (*P* < .05). In addition, 5 studies^[16,18,22,28,34]^ (514 patients), 4 studies^[18,22,28,34]^ (424 patients) and 4 studies^[18,22,28,34]^ (424 patients) respectively investigated the dose-effect of TXA (low *versus* medium, low vs high, medium vs high) on total volume of postoperative bleeding in adults. Meta-analysis suggested that, both medium- and high-dose TXA are more effective than low-dose TXA in total blood loss reduction (*P* < .05). However, total postoperative bleeding volume of medium- and high-dose TXA were comparable (*P* > .05).

For pediatrics, 4 studies^[32,35–37]^ (3620 patients) compared total postoperative blood loss of TXA versus Control, and meta-analysis demonstrated that, TXA remarkably reduced postoperative blood loss as compared to Control (Fig. 3). As shown in Table S2, Supplemental Digital Content, http://links.lww.com/MD/J6, TXA also significantly reduced the incidences of postoperative massive bleeding and reoperation for bleeding, shortened postoperative chest drainage duration in adult patients (*P* < .05).

### 3.5. Effects on postoperative blood transfusion

As shown in Table S2, Supplemental Digital Content, http://links.lww.com/MD/J6, TXA administration significantly reduced postoperative transfusion incidences and volume of both allogenic RBC and FFP, and reduced postoperative transfusion incidence of PC (*P* < .05) without obvious dose-effects (*P* > .05), but TXA did not reduce PC transfusion volume postoperatively in adult patients (*P* > .05). For pediatric patients, TXA did not significantly reduce postoperative transfusion incidence and volume of allogenic RBC, FFP and PC as compared to Control (*P* > .05).

### 3.6. Effects on postoperative recovery

As shown in Table S3, Supplemental Digital Content, http://links.lww.com/MD/J7, TXA administration did not affect postoperative Mechanical ventilation duration or LOS in the intensive care unit of both adult and pediatric patients. Ten studies^[15,19–21,23–26,29,30]^ (1750 patients) demonstrated that TXA shorten postoperative LOS in the hospital as compared to Control in adults (*P* < .05). Two studies^[37,38]^ (1564 patients) reported that TXA had no effect on hospitalization expenditures in pediatric patients (*P* > .05).

### 3.7. Effects on mortality and morbidities during hospitalization

With respect to the composite incidence of postoperative mortality and mortalities during hospitalization of adult patients, 14 studies^[15,16,18–21,23–27,29–31]^ (2384 patients), 3 studies^[16–18]^ (195 patients),1 study^[18]^ (64 patients) investigated the effects of low-, medium- and high-dose TXA as compared to Control, respectively (Fig. 4). Meta-analysis demonstrated that, the composite rate of postoperative mortality and morbidities during hospitalization of adult patients were comparable among low-, medium-, high-dose TXA and Control groups, and that there was no obvious dose-effect of TXA. Also as shown in Figure 4, TXA had no influence on the composite rate of postoperative mortality and morbidities in pediatric patients (*P* > .05).

### 3.8. Effects on postoperative seizure during hospitalization

As shown in Figure S3, Supplemental Digital Content, http://links.lww.com/MD/J8, 4 studies^[15,21,25,27]^ (1155 patients) and 1 study^[35]^ (2026 patients) reported the incidence of seizure during hospitalization in adult and pediatric patients, respectively. Meta-analysis demonstrated that, TXA did not increase the risk of postoperative seizure during hospitalization as compared to Control either in adults or in pediatrics (*P* > .05). Three studies^[22,28,34]^ (358 patients) compared the dose-effects of TXA on the incidence of postoperative seizure in adult patients. Meta-analysis suggested that, the incidences of postoperative seizure among low-, medium- and high-dose of TXA groups were comparable (*P* > .05).

### 3.9. Effects on mortality and morbidities during follow-ups

As shown in Figure S4, Supplemental Digital Content, http://links.lww.com/MD/J9, 3 studies^[22,25,27]^ (871 patients), 1 study^[21]^ (202 patients), 1 study^[21]^ (202 patients), 2 studies^[36,37]^ (390 patients), 1 study^[37]^ (386 patients) reported mortality and morbidities during 1-, 3-, 5-, 7-, and 8-year follow-ups, respectively. Meta-analysis demonstrated that, TXA was not associated with increased composite incidence of long-term mortality and morbidities postoperatively as compared to Control (*P* > .05).

### 3.10. Effects on coagulation and fibrinolytic functions

As shown in Table S4, Supplemental Digital Content, http://links.lww.com/MD/J10, 5 studies^[21,23–26]^ (973 patients) reported intraoperative heparin dose, 6 studies^[15,21,23–26]^ (1257 patients) reported protamine dose for heparinization reversal, 6 studies^[15,21,23–26]^ (1257 patients) reported protamine: heparin dose ratio, and 1 study^[29]^ (231 patients) reported activated clotting time values at the end of operation in adults. Meta-analysis demonstrated that, TXA administration had no effects on intraoperative heparinization and its reversal (*P* > .05). TXA did not affect prothrombin time (PT), Plt count and function postoperatively, either (*P* > .05). However, TXA was associated with higher postoperative PT-international normalized ratio (INR) (*P* < .05), especially when comparing high- versus low-dose TXA and high- *versus* medium dose (*P* < .05) (Table S4, Supplemental Digital Content, http://links.lww.com/MD/J10). As shown in Table S4, Supplemental Digital Content, http://links.lww.com/MD/J10, TXA significantly reduced D-dimer level 24 hours postoperatively (*P* < .05), especially when comparing high-dose TXA *versus* low-dose TXA and high-dose TXA *versus* medium dose TXA (*P* < .05).

### 3.11. Effects on inflammation, myocardial and renal biomarkers

As shown in Table S5, Supplemental Digital Content, http://links.lww.com/MD/J11, 1 study^[16]^ (139 patients) reported WBC count, and demonstrated that TXA administration had no effects on postoperative WBC count. One study^[30]^ (60 patients), 1 study^[19]^ (40 patients) and 1 study^[16]^ (60 patients) investigated the effects of TXA on the levels of IL-6, Polymorphonuclear neutrophil elastase (PMNE) and fibronectin respectively, and demonstrated that TXA significantly reduced the release of IL-6 and PMNE, but increased the level of fibronectin postoperatively.^[16]^ One study^[31]^ (140 patients) demonstrated that postoperative cTn I levels of TXA and Control groups were similar (*P* > .05), but creatine kinase-MB level was significantly lower in TXA group (*P* < .05). One study^[29]^ (231 patients) demonstrated that postoperative creatinine levels of TXA and Control groups were comparable (*P* > .05).

## 4. Discussion

TXA reduces bleeding by several mechanisms, including prevention of plasminogen-fibrin binding, inhibition of plasmin-induced Plt activation and enhanced Plt adenosine diphosphate and arachidonic concentration, stabilization of clot and attenuation of systemic inflammatory response.^[5,9]^ The current study demonstrated that, intravenous TXA reduced postoperative blood loss in both adult and pediatric patients undergoing cardiac surgery. The beneficial effects of TXA on surgical blood loss have been previously proved in several types of cardiac surgeries (e.g., CABG,^[38,39]^ valvular surgery^[40]^ and pediatric cardiac surgery^[41]^). Nevertheless, whether TXA has different effects on different subgroups of cardiovascular surgical patients (e.g., ages, surgical procedures, renal function) and its optimal dosages warrant further investigation.^[6–9,42,43]^

Similar to previous findings,^[6,44]^ the present study demonstrated that, intravenous TXA significantly reduced postoperative transfusion requirement of RBC, FFP, and PC in adult patients. However, the present study demonstrated that TXA did not reduce postoperative transfusion incidence and volume of allogenic RBC, FFP and PC in pediatrics, which is also in agreement with the findings of our recent meta-analysis of 15 RCTs investigating the effects and safety of TXA in Chinese (Asian) pediatric patients undergoing cardiac surgery.^[45]^ In contrast, a previous meta-analysis of 8 RCTs evaluating the efficacy of TXA in Caucasian pediatric cardiac surgical patients suggested that TXA did not reduce postoperative blood loss, but reduced the need of transfusion of RBC, FFP and PC.^[43]^ The inconsistent findings are likely caused by different races of enrolled patients. Evidence has suggested that there are significant differences among different human races with respect to coagulation and fibrinolysis functions.^[46–48]^

The present study demonstrated that intravenous TXA did not influence the composite incidence of mortality and morbidities in either adults or pediatrics during hospitalization, and there was no obvious dose-effect of TXA in adult patients. In 2007, the Blood conservation using Antifibrinolytics in a randomized Trial study, reported an increased 30-day mortality in high-risk cardiac surgical patients associated with aprotinin (6%) as compared to lysine analogs TXA (3.9%) and epsilon aminocaproic acid (4%).^[49]^ More recently, a large-volume multicentral study revealed that among cardiac surgical patients high-dose compared with low-dose tranexamic acid infusion met criteria for noninferiority with respect to a composite primary safety end point consisting of 30-day mortality, seizure, kidney dysfunction, and thrombotic events.^[50]^ The mechanisms of TXA on mortality in cardiac and noncardiac surgical patients and its dose-effect profiles need to be scrutinized in more well-designed studies. TXA-associated thrombotic complications (e.g., stroke, myocardial infarction, deep vein thrombosis/pulmonary embolism, renal insufficiency, bowl infarction) have also been a concern. For example, it has been reported that intraoperative TXA administration was significantly associated with increased risk of postoperative stroke in patients undergoing cardiac surgery by us and others.^[1,51]^ Currently, it remains unknown whether TXA has any beneficial or benevolent effect on myocardial infarction or ischemia *via* plasmin inhibition or other mechanisms.^[21,31]^ Preliminary evidence has suggested a possible association of TXA administration and an increased risk of acute myocardial infarction, even in patients with relatively low thrombotic risk.^[52]^ The retrospective analysis from Fuwai Hospital also demonstrated that TXA administration led to increased risk of perioperative myocardial infarction by1.4-fold.^[33]^ Intriguingly, the effect of TXA on myocardial biomarkers (e.g., cTn, creatine kinase-MB) in cardiac surgical patients remains controversial.^[31,53–56]^ At present, the experimental evidence remains scarce concerning the effects of TXA on myocardial ischemia reperfusion injury. A previous animal experiment utilizing in vivo model of left anterior descending coronary artery occlusion and reperfusion demonstrated that, TXA neither directly influenced myocardial infarction size nor influenced infarction size reduction by ischemia preconditioning or remote ischemia preconditioning.^[57]^ It is also noteworthy that, the effect of TXA on other organs (e.g., brain, lung, kidney, liver, intestines) has received little attention.

TXA-related seizure has been another concern for decade.^[58–60]^ Seizure is transient occurrence of signs and/or symptoms due to abnormal excessive or synchronous neuronal activity in the brain, which increases the risk of neuronal injury and neuronal networks alteration.^[61,62]^ Seizure after cardiac surgery could be contributed to multiple risk factors [e.g., advanced patient age, preexisting neurologic diseases, renal dysfunction, open heart surgery, lengthy cardiopulmonary bypass (CPB), and high-dose TXA].^[62,63]^ The proconvulsive property of TXA might be elicited by its direct effect on neurons and glia cells.^[63]^ TXA can cross the blood-brain barrier easily, thus it not only acts as a competitive antagonist at the glycine receptors,^[63]^ but also facilitates neuronal excitation by antagonizing the inhibitory effect of GABA-ergic neurotransmission.^[64]^ The reported incidence of postoperative seizure at Fuwai Hospital was between 0.7% and 1.1%.^[65]^ It is noteworthy that the diagnosis of TXA-induced seizure of previous studies was often made clinically without electroencephalogram confirmation. Currently, there is an ongoing large-scale TXA RCT (NCT03782350)^[66]^ at Fuwai Hospital, which utilized electroencephalogram monitoring to assess the effects of TXA dose on the occurrence of postoperative seizure.

The current study also indicated that, TXA was not associated with increased composite incidence of long-term mortality and mortalities up to 8 years postoperatively. Similarly, recent large RCT by Myles et al^[67]^ which enrolled 4631 patients undergoing CABG demonstrated that, TXA reduced bleeding and blood transfusion without increased thrombotic complications or death up to 1 year postoperatively. As previously pointed out, long-term mortality and morbidities of cardiac surgical patients most likely represents the natural process of aging and pathologic developments of co-existing diseases.^[68]^ Whether single intraoperative administration of TXA has any long-term effects on mortality and morbidities need further investigation.

Similar to previous report,^[44]^ the present study suggested that TXA did not influence intraoperative heparin/protamine dosages, and did not influence activated clotting time values after heparin reversal at the end of surgery, either. Contrary to previous findings that TXA lowered postoperative PT values and INR in patients undergoing valvular surgery,^[69]^ the present study demonstrated that TXA was associated with higher postoperative PT-INR and the reason remains unknown. D-dimer, the degradation product resulting from plasmin-induced clot lysis, whose levels are used as a marker for fibrinolysis. Elevated levels of D-dimer are often indicative of hyperfibrinolysis and hemorrhagic risk in cardiac surgical patients. The present study indicated that, TXA significantly decreased D-dimer levels postoperatively. Thrombocytopenia and Plt dysfunction are consequences of hemodilution and hypothermia during CPB, which also significantly increase the risk of bleeding complications and transfusion requirements. Contrary to previous studies reporting that TXA preserved or improved Plt dysfunction induced by cardiac surgery and CPB,^[70,71]^ the present study demonstrated that TXA did not affect postoperative Plt count and function. The reason could be partially explained by that majority of the 23 studies excluded patients with continued preoperative antiplatelet therapy.

The optimal perioperative antiplatelet strategy for patients with acute coronary syndrome requiring surgical revascularization remains unclear because of competing risks of myocardial ischemic events and bleeding. Aspirin inhibits Plt function by selectively depressing cyclooxygenase and interrupting thromboxane A2 formation. Clopidogrel is a thienopyridine inhibiting adenosine diphosphate dependent triggered Plt activation and aggregation. Aspirin and clopidogrel are used singularly single antiplatelet therapy (SAPT) or in combination dual antiplatelet therapy (DAPT).^[6]^ Both SAPT and DAPT increase the risk of excessive bleeding and transfusion. DAPT even causes synergistic inhibition of thromboxane A2 and adenosine diphosphate dependent-dependent Plt activation pathways.^[72]^ In addition to its antifibrinolytic effect, TXA could also increase Plt aggregation in with aspirin or/and clopidogrel treated patients, and reduce bleeding or transfusion exposure in cardiac surgical patients with continued SAPT and DAPT.^[24–27,72–75]^

Systemic inflammatory response syndrome is induced in nearly all patients undergoing cardiac surgery.^[76]^ Excessive plasmin activity and/or D-dimer formation play an important role in pro-inflammatory cytokines and cellular response activation.^[77]^ The current study also suggested that TXA elicited anti-inflammatory effects. Consistent with that, a recent meta-analysis by us evaluating the effect of TXA on inflammatory biomarkers in adult cardiac surgical patients, demonstrated that TXA has significant anti-inflammatory effects as manifested by reduction of multiple pro-inflammatory mediators (e.g., IL-6, tumor necrosis factor-α, IL-1β, PMNE).^[78]^

There are several limitations worth to be mentioned. Firstly, most data of the present study were homogeneous due to similar investigators, clinical care protocols and outcome definitions. Even so, the present study was biased because it was a *pos hoc* analysis of clinical studies performed at a single center. Therefore, the conclusions should be explained with caution and could not be straightly extrapolated. Secondly, all pediatric patients of the present study were retrospectively enrolled. Therefore, the benefit-to-risk ratio associated with TXA in pediatric cardiac surgical patients cannot be adequately defined yet.^[41,45,79]^ Thirdly, the dose regimen of TXA varied among some included studies. Evidence has suggested that both the blood-saving effects of TXA and its side effects (e.g., seizure) are dose-dependent.^[6–10]^ Therefore, the optimal dosages of TXA in different subgroups of cardiovascular surgical patients are urgently warranted.^[80,81]^

To conclude, this current study suggested that intravenous TXA significantly reduced total volume of postoperative bleeding in both adult and pediatric patients undergoing cardiac surgery at the single cardiovascular center without increasing the composite incidence of mortality and morbidities.

## Acknowledgements

The authors were so grateful to Miss Xiao Han, Dr Hua Ying and Dr Shunzi Peng-Ri for their help during the process of the present work.

## Author contributions

**Conceptualization:** Yuntai Yao.

**Data curation:** Peishuang Lin, Yuntai Yao.

**Methodology:** Peishuang Lin, Yuntai Yao, Lijuan Tian, Juanjuan Jiang, Yang Zhang, Lixian He, Yiping Yu, Jie Ma.

**Software:** Yuntai Yao, Lijuan Tian.

**Validation:** Peishuang Lin, Juanjuan Jiang, Yang Zhang, Lixian He, Yiping Yu, Jie Ma.

**Visualization:** Yuntai Yao.

**Writing – original draft:** Yuntai Yao.

## Supplementary Material
