# Supplementary material for: The efficacy and safety of intravenous administration of tranexamic acid in patients undergoing cardiac surgery: Evidence from a single cardiovascular center
Source: Medicine (Baltimore). 2023 May 17;102(20):e33819. doi: 10.1097/MD.0000000000033819 (PMC10194539; doi:10.1097/MD.0000000000033819)

Supplement Figure 1. Risk of bias summary

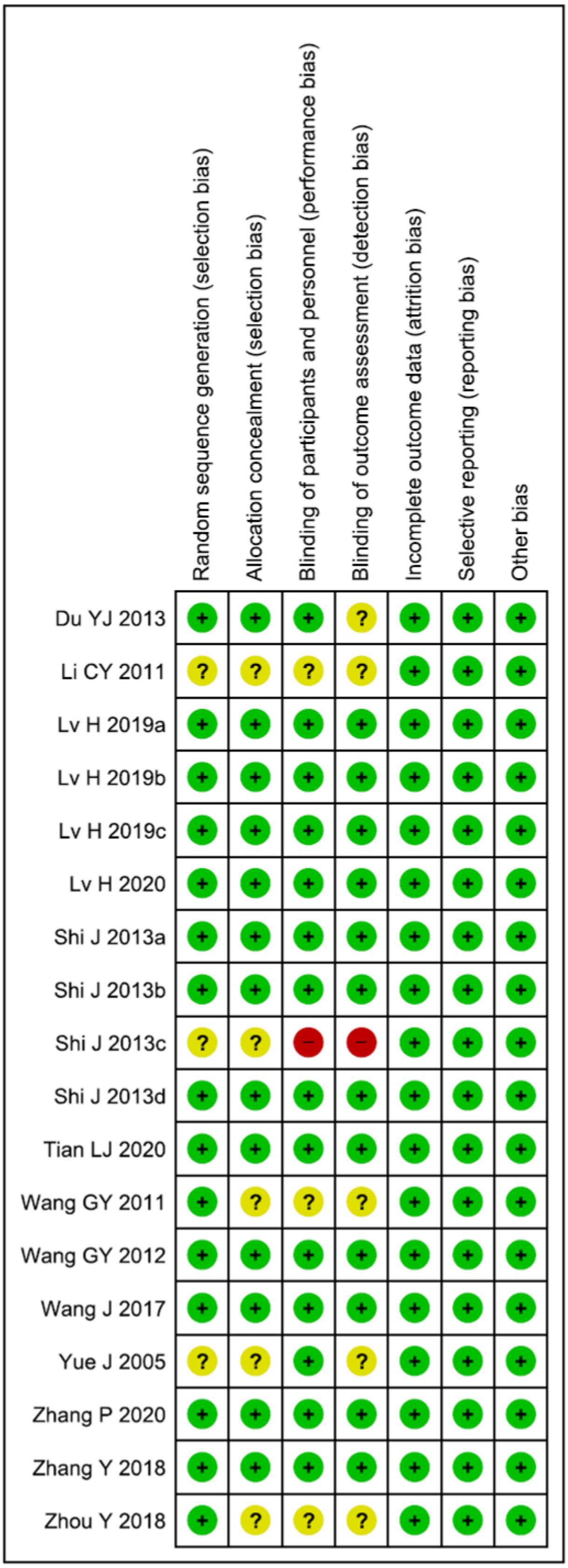

Supplement Figure 2. Risk of bias graph

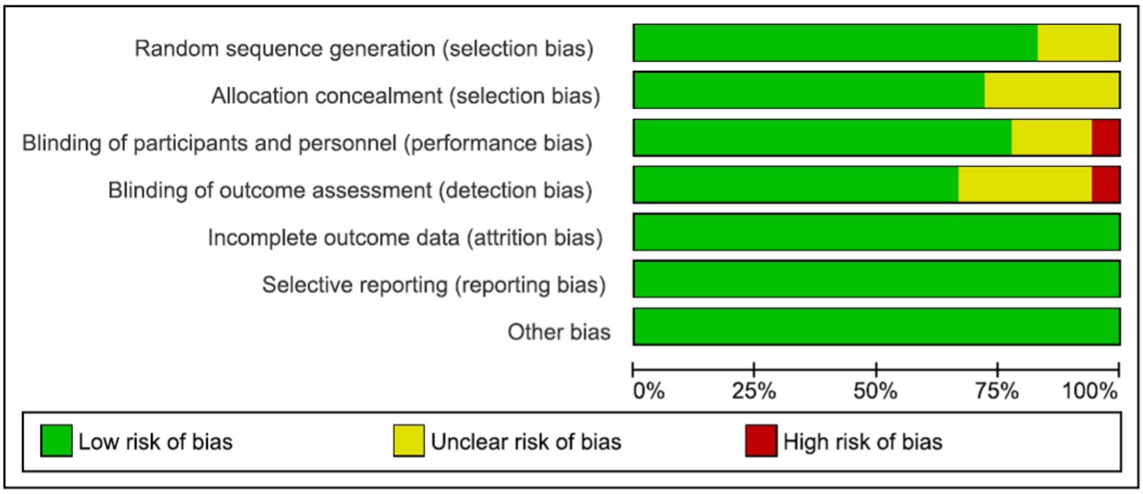

Supplement: Supplementary file 1 [file medi-102-e33819-s001.pdf]
