# Supplementary material for: The efficacy and safety of intravenous administration of tranexamic acid in patients undergoing cardiac surgery: Evidence from a single cardiovascular center
Source: Medicine (Baltimore). 2023 May 17;102(20):e33819. doi: 10.1097/MD.0000000000033819 (PMC10194539; doi:10.1097/MD.0000000000033819)
Supplement: Supplementary file 3 [file medi-102-e33819-s003.pdf]

Supplement Table 2. Post-operative bleeding and transfusion

Outcomes	Studies(n)	Patients(n)	Heterogeneity *P* value	I^2	WMD	OR	95%CI	Overall effect *P* value
**Post-op massive bleeding**(%)								
①*Adults*								
TXA vs. CTRL	8	1,585	0.90	0%	-	0.44	0.36, 0.54	<0.0001*
TXA(LD) vs. TXA(MD)	2	162	0.73	0%	-	1.30	0.69, 2.47	0.42
TXA(LD) vs. TXA(HD)	2	164	0.74	0%	-	1.91	0.99, 3.70	0.05
TXA(MD) vs. TXA(HD)	2	164	0.51	0%	-	1.47	0.75, 2.87	0.26
②*Pediatrics*								
TXA vs. CTRL	0							
**Post-op re-operation for bleeding**(%)								
①*Adults*								
TXA vs. CTRL	11	22,854	0.15	15%	-	0.71	0.59, 0.86	0.0003*
TXA(LD) vs. TXA(MD)	3	262	0.84	0%	-	2.06	0.37, 11.47	0.41
TXA(LD) vs. TXA(HD)	3	263	0.83	0%	-	2.09	0.38, 11.57	0.40
TXA(MD) vs. TXA(HD)	3	265	NA	NA	-	0.98	0.06, 16.11	0.99
②*Pediatrics*								
TXA vs. CTRL	2	3,262	0.64	0%	-	0.55	0.14, 2.19	0.39
**Chest drainage duration**(h)								
①*Adults*								
TXA vs. CTRL	8	1,459	0.89	0%	-	-2.13	-3.80, -0.47	0.01*
②*Pediatrics*								
TXA vs. CTRL	0							
**Any transfusion**(%)								
①*Adults*								

TXA *vs.* CTRL	13	22,763	0.18	25%	-	0.45	0.42, 0.48	<0.00001*
TXA(LD) *vs.* TXA(MD)	2	162	0.72	0%	-	1.64	0.78, 3.46	0.19
TXA(LD) *vs.* TXA(HD)	2	164	0.74	0%	-	1.49	0.70, 3.14	0.30
TXA(MD) *vs.* TXA(HD)	2	164	0.98	0%	-	0.91	0.45, 1.82	0.78
②*Pediatrics*								
TXA vs. CTRL	0							
**RBC transfusion**(%)								
①*Adults*								
TXA *vs.* CTRL	15	23,432	0.93	0%	-	0.51	0.48, 0.54	<0.00001*
TXA(LD) *vs.* TXA(MD)	4	354	0.40	0%	-	1.36	0.89, 2.09	0.16
TXA(LD) *vs.* TXA(HD)	3	263	0.70	0%	-	1.14	0.69, 1.88	0.62
TXA(MD) *vs.* TXA(HD)	3	265	0.66	0%	-	0.85	0.51, 1.38	0.51
②*Pediatrics*								
TXA *vs.* CTRL	3	3,590	0.03(R)	73%	-	0.89	0.63, 1.27	0.53
**RBC transfusion**(unit)								
①*Adults*								
TXA *vs.* CTRL	13	2,334	0.005	53%	-2.30	-	-2.85, -1.74	<0.00001*
TXA(LD) *vs.* TXA(MD)	3	356	0.81	0%	0.21	-	-0.46, 0.87	0.54
TXA(LD) *vs.* TXA(HD)	3	299	0.74	0%	0.26	-	-0.65, 1.18	0.57
TXA(MD) *vs.* TXA(HD)	3	265	0.84	0%	-0.17	-	-0.96, 0.63	0.68
②*Pediatrics*								
TXA *vs.* CTRL	3	3,590	<0.00001	100%	-0.05	-	-0.20, 0.09	0.48
**FFP transfusion**(%)								
①*Adults*								
TXA *vs.* CTRL	14	23,093	0.57	0%	-	0.40	0.37, 0.42	<0.00001*

TXA(LD) *vs.* TXA(MD)	4	354	0.96	0%	-	1.45	0.93, 2.27	0.10
TXA(LD) *vs.* TXA(HD)	3	263	0.51	0%	-	1.12	0.67, 1.87	0.67
TXA(MD) *vs.* TXA(HD)	3	265	0.79		-	0.80	0.48, 1.35	0.41
②*Pediatrics*								
TXA *vs.* CTRL	3	3,590	0.001	85%	-	1.01	0.65, 1.55	0.98
**FFP transfusion**(ml)								
①*Adults*								
TXA *vs.* CTRL	12	2,334	0.04	41%	-194.19	-	-223.45, -164.93	<0.00001*
TXA(LD) *vs.* TXA(MD)	4	356	0.99	0%	4.00	-	-88.09, 96.10	0.93
TXA(LD) *vs.* TXA(HD)	3	265	0.27	25%	13.61	-	-90.69, 117.91	0.80
TXA(MD) *vs.* TXA(HD)	3	265	0.50	0%	3.11	-	-112.23, 118.46	0.96
②*Pediatrics*								
TXA *vs.* CTRL	3	3,590	<0.00001	100%	-5.01	-	-11.35, 1.32	0.12
**PC transfusion**(%)								
①*Adults*								
TXA *vs.* CTRL	13	23,033	0.02	47%	-	0.66	0.56, 0.78	<0.00001*
TXA(LD) *vs.* TXA(MD)	3	154	NA	NA	-	1.04	0.06, 17.13	0.98
TXA(LD) *vs.* TXA(HD)	2	163	NA	NA	-	0.33	0.03, 3.25	0.34
TXA(MD) *vs.* TXA(HD)	2	165	NA	NA	-	0.31	0.03, 3.12	0.32
②*Pediatrics*								
TXA *vs.* CTRL	3	3,590	0.55	0%	-	1.95	0.92, 4.11	0.08
**PC transfusion**(unit)								
①*Adults*								
TXA *vs.* CTRL	11	1,894	0.85	0%	-0.03	-	-0.07, 0.01	0.20
TXA(LD) *vs.* TXA(MD)	4	356	NA	NA	0.00	-	-0.05, 0.05	1.00

TXA(LD) *vs.* TXA(HD)	3	265	NA	NA	-0.06	-	-0.16, 0.04	0.25
TXA(MD) *vs.* TXA(HD)	3	265	NA	NA	-0.06	-	-0.16, 0.04	0.25
②*Pediatrics*								
TXA *vs.* CTRL	3	3,590	<0.00001	100%	0.02	-	-0.00, 0.05	0.10

CI=confidence interval, CTRL=control, FFP=fresh frozen plasma, HD=high dose, Intra-op=intra-operative, LD=low dose, MD=medium dose, NE=not estimable, NA=not applicable, OR=odds ratio, PC=platelet concentrate, Post-op=post-operative, RBC=red blood cell, TXA=tranexamic acid, WMD=weighted mean difference.
