# Supplementary material for: The efficacy and safety of intravenous administration of tranexamic acid in patients undergoing cardiac surgery: Evidence from a single cardiovascular center
Source: Medicine (Baltimore). 2023 May 17;102(20):e33819. doi: 10.1097/MD.0000000000033819 (PMC10194539; doi:10.1097/MD.0000000000033819)
Supplement: Supplementary file 4 [file medi-102-e33819-s004.pdf]

Supplement Table 3. Post-operative recovery

Outcomes	Studies(n)	Patients(n)	Heterogeneity *P* value	I^2	WMD	OR	95%CI	Overall effect *P* value
**Post-op MVD**(h)								
①*Adults*								
TXA *vs.* CTRL	9	1,657	0.50	0%	0.16	-	-032, 0.64	0.51
TXA(LD) *vs.* TXA(MD)	4	354	0.48	0%	1.81	-	0.18, 3.44	0.03*
TXA(LD) *vs.* TXA(HD)	3	263	0.79	0%	-0.38	-	-2.73, 1.96	0.74
TXA(MD) *vs.* TXA(HD)	3	263	0.70	0%	-0.97	-	-3.27, 1.33	0.41
②*Pediatrics*								
TXA *vs.* CTRL	2	1,564	0.02(R)	83%	1.25	-	-1.65, 4.15	0.40
**Post-op LOS in the ICU**(h)								
①*Adults*								
TXA *vs.* CTRL	10	1,750	0.70	0%	0.42	-	-1.61, 2.45	0.69
TXA(LD) *vs.* TXA(MD)	4	354	0.48	0%	3.65	-	-3.04, 10.34	0.28
TXA(LD) *vs.* TXA(HD)	3	263	0.72	0%	0.49	-	-7.53, 8.52	0.90
TXA(MD) *vs.* TXA(HD)	3	263	0.17	44%	-3.12	-	-10.80, 4.56	0.43
②*Pediatrics*								
TXA *vs.* CTRL	3	3,590	1.00	0%	0.00	-	-1.15, 1.15	1.00
**Post-op LOS in the hospital**(d)								
①*Adults*								
TXA *vs.* CTRL	10	1,750	0.79	0%	-0.30	-	-0.48, -0.11	0.002*
TXA(LD) *vs.* TXA(MD)	4	354	0.81	0%	-0.69	-	-1.55, 0.17	0.11
TXA(LD) *vs.* TXA(HD)	3	263	0.96	0%	0.90	-	-0.30, 2.10	0.14
TXA(MD) *vs.* TXA(HD)	3	263	0.89	0%	1.07	-	-0.30, 2.45	0.13
②*Pediatrics*								

TXA *vs.* CTRL	3	3,590	0.53	0%	0.01	-	-0.07, 0.09	0.80
**Hospitalization expenditures**(×10^4 Chinese Yuan)								
①*Adults*								
TXA *vs.* CTRL	0							
②*Pediatrics*								
TXA *vs.* CTRL	2	1,564	0.89	0%	0.06	-	-0.01, 0.13	0.10

CI=confidence interval, CTRL=control, HD=high dose, EOS=end of surgery, ICU=intensive care unit, LD=low dose, LOS=length of stay, MD=medium dose, OR=odds ratio, Post-op=post-operative, MVD=mechanical ventilation duration, TXA=tranexamic acid, WMD=weighted mean difference.
