# Supplementary material for: The efficacy and safety of intravenous administration of tranexamic acid in patients undergoing cardiac surgery: Evidence from a single cardiovascular center
Source: Medicine (Baltimore). 2023 May 17;102(20):e33819. doi: 10.1097/MD.0000000000033819 (PMC10194539; doi:10.1097/MD.0000000000033819)

Supplement Figure 3. Forest plot of the incidence of postoperative seizure in adults and pediatrics during hospitalization

	Control or Lower-Dose TXA		TXA or Higher-Dose TXA			Odds Ratio
Study or Subgroup	Events	Total	Events	Total	Weight	M-H, Random, 95% CI
**2.1.1 Hospitalization seizure in adults(%): Control vs. Low-Dose TXA**						
Shi J 2013b	0	59	1	58	13.4%	0.32 [0.01, 8.07]
Shi J 2013d	2	274	4	278	47.8%	0.50 [0.09, 2.77]
Zhang P 2020	1	141	2	143	23.9%	0.50 [0.05, 5.62]
Zhang Y 2018d	0	102	2	100	14.9%	0.19 [0.01, 4.05]
**Subtotal (95% CI)**		**576**		**579**	**100.0%**	**0.41 [0.13, 1.34]**
Total events	3		9			
Heterogeneity: Tau² = 0.00; Chi² = 0.35, df = 3 (P = 0.95); I² = 0%						
Test for overall effect: Z = 1.48 (P = 0.14)						
**2.1.2 Hospitalization seizure in adults(%): Control vs. Medium-Dose TXA**						
**Subtotal (95% CI)**		**0**		**0**		**Not estimable**
Total events	0		0			
Heterogeneity: Not applicable						
Test for overall effect: Not applicable						
**2.1.3 Hospitalization seizure in adults(%): Control vs. High-Dose TXA**						
**Subtotal (95% CI)**		**0**		**0**		**Not estimable**
Total events	0		0			
Heterogeneity: Not applicable						
Test for overall effect: Not applicable						
**2.1.4 Hospitalization seizure in adults(%): Low vs. Medium-Dose TXA**						
Du YJ 2013(1)	1	49	0	51	100.0%	3.19 [0.13, 80.09]
Liu Y 2021(1)	0	80	0	78		Not estimable
Zhou Y 2018(1)	0	50	0	50		Not estimable
**Subtotal (95% CI)**		**179**		**179**	**100.0%**	**3.19 [0.13, 80.09]**
Total events	1		0			
Heterogeneity: Not applicable						
Test for overall effect: Z = 0.70 (P = 0.48)						
**2.1.5 Hospitalization seizure in adults(%): Low vs. High-Dose TXA**						
Du YJ 2013(2)	1	49	1	50	56.9%	1.02 [0.06, 16.79]
Liu Y 2021(2)	0	80	1	79	43.1%	0.33 [0.01, 8.10]
Zhou Y 2018(2)	0	50	0	50		Not estimable
**Subtotal (95% CI)**		**179**		**179**	**100.0%**	**0.62 [0.08, 5.15]**
Total events	1		2			
Heterogeneity: Tau² = 0.00; Chi² = 0.28, df = 1 (P = 0.60); I² = 0%						
Test for overall effect: Z = 0.44 (P = 0.66)						
**2.1.6 Hospitalization seizure in adults(%): Medium vs. High-Dose TXA**						
Du YJ 2013(3)	1	50	0	51	49.9%	3.12 [0.12, 78.45]
Liu Y 2021(3)	1	79	0	78	50.1%	3.00 [0.12, 74.77]
Zhou Y 2018(3)	0	50	0	50		Not estimable
**Subtotal (95% CI)**		**179**		**179**	**100.0%**	**3.06 [0.31, 29.82]**
Total events	2		0			
Heterogeneity: Tau² = 0.00; Chi² = 0.00, df = 1 (P = 0.99); I² = 0%						
Test for overall effect: Z = 0.96 (P = 0.34)						
**2.1.7 Hospitalization seizure in pediatrics(%): Control vs. TXA**						
Zhang Y 2019a	0	1056	0	970		Not estimable
**Subtotal (95% CI)**		**1056**		**970**		**Not estimable**
Total events	0		0			
Heterogeneity: Not applicable						
Test for overall effect: Not applicable						

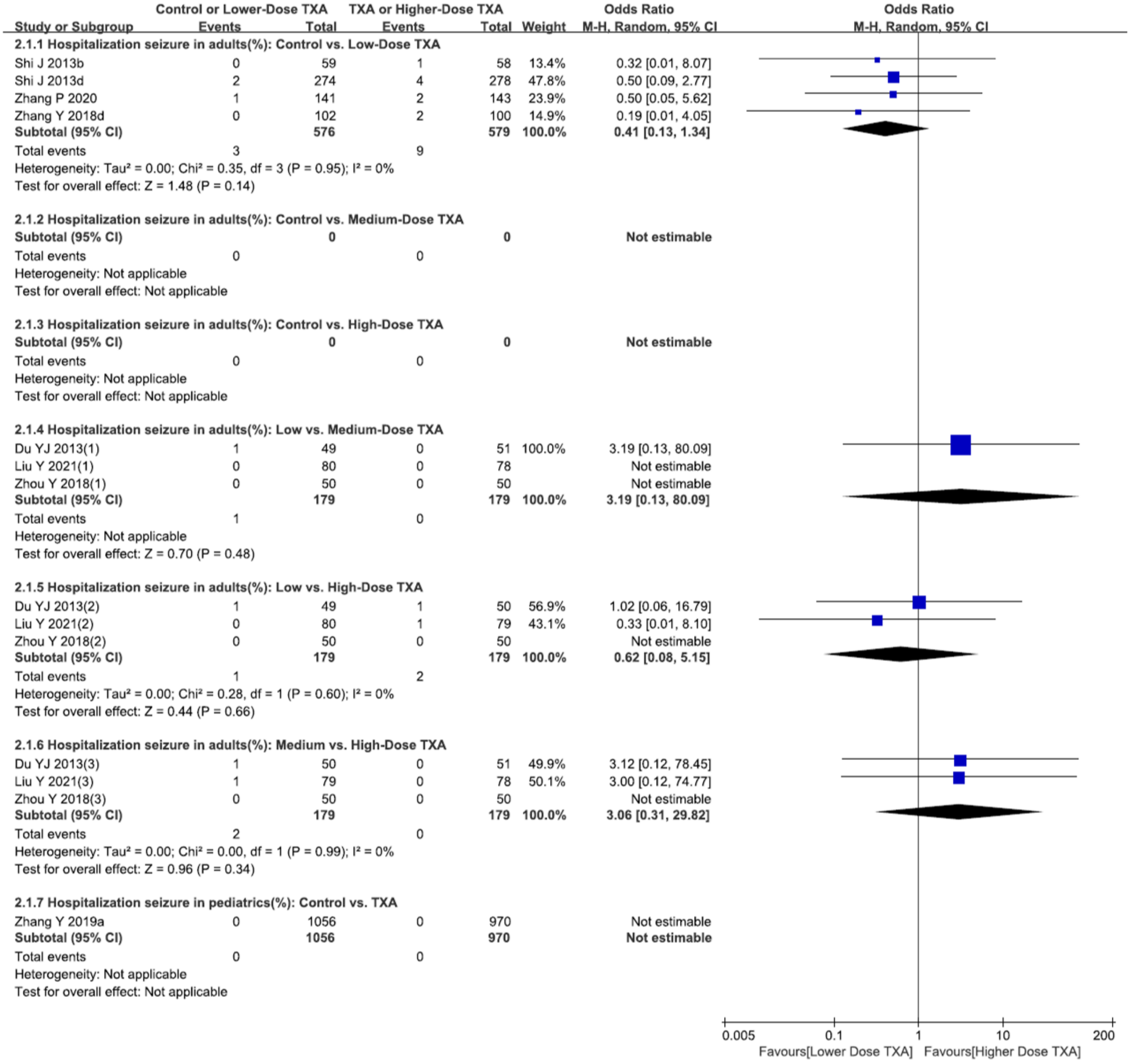

Supplement: Supplementary file 5 [file medi-102-e33819-s005.pdf]
