# Supplementary material for: The efficacy and safety of intravenous administration of tranexamic acid in patients undergoing cardiac surgery: Evidence from a single cardiovascular center
Source: Medicine (Baltimore). 2023 May 17;102(20):e33819. doi: 10.1097/MD.0000000000033819 (PMC10194539; doi:10.1097/MD.0000000000033819)

Supplement Figure 4. Forest plot of the composite incidence of mortality and morbidities in adults and pediatrics during long-term follow-ups

Study or Subgroup	Control Events	Control Total	TXA Events	TXA Total	Weight	Odds Ratio M-H, Random, 95% CI
**1.43.1 Hospitalization mortality and morbidities(%)**						
Li CY 2011	0	70	0	70		Not estimable
Lv H 2019a(1)	0	10	0	31		Not estimable
Lv H 2019a(2)	0	11	0	31		Not estimable
Lv H 2019a(3)	0	11	0	33		Not estimable
Lv H 2019b	0	53	0	48		Not estimable
Lv H 2019c	0	50	0	51		Not estimable
Lv H 2020	0	20	0	20		Not estimable
Shi J 2013a	2	55	3	55	8.0%	0.65 [0.10, 4.08]
Shi J 2013b	4	59	4	58	12.9%	0.98 [0.23, 4.13]
Shi J 2013c(1)	0	60	0	60		Not estimable
Shi J 2013d	11	274	9	278	33.1%	1.25 [0.51, 3.07]
Tian LJ 2020(1)	0	23	0	46		Not estimable
Tian LJ 2020(2)	0	24	0	46		Not estimable
Wang ES 2021	896	10519	1098	10519	0.0%	0.80 [0.73, 0.88]
Wang GY 2011	0	30	0	30		Not estimable
Wang GY 2012	0	115	0	116		Not estimable
Wang J 2017	2	106	0	105	2.9%	5.05 [0.24, 106.42]
Zhang P 2020	9	141	7	143	25.8%	1.32 [0.48, 3.66]
Zhang Y 2018a	1	138	1	190	3.4%	1.38 [0.09, 22.25]
Zhang Y 2018b	0	648	1	588	2.6%	0.30 [0.01, 7.43]
Zhang Y 2018d	2	102	2	100	6.8%	0.98 [0.14, 7.10]
Zhang Y 2019a	2	1056	1	970	4.6%	1.84 [0.17, 20.31]
**Subtotal (95% CI)**		**3056**		**3069**	**100.0%**	**1.18 [0.70, 1.97]**
Total events	33		28			
Heterogeneity: Tau² = 0.00; Chi² = 2.28, df = 8 (P = 0.97); I² = 0%						
Test for overall effect: Z = 0.62 (P = 0.54)						
**1.43.2 1-year postoperative mortality and morbidities(%)**						
Shi J 2013b	7	59	6	58	28.9%	1.17 [0.37, 3.71]
Shi J 2013d	13	278	14	274	64.5%	0.91 [0.42, 1.98]
Zhang Y 2018d	2	100	1	102	6.6%	2.06 [0.18, 23.10]
**Subtotal (95% CI)**		**437**		**434**	**100.0%**	**1.03 [0.55, 1.92]**
Total events	22		21			
Heterogeneity: Tau² = 0.00; Chi² = 0.46, df = 2 (P = 0.80); I² = 0%						
Test for overall effect: Z = 0.10 (P = 0.92)						
**1.43.3 3-year postoperative mortality and morbidities(%)**						
Zhang Y 2018d	2	100	1	102	100.0%	2.06 [0.18, 23.10]
**Subtotal (95% CI)**		**100**		**102**	**100.0%**	**2.06 [0.18, 23.10]**
Total events	2		1			
Heterogeneity: Not applicable						
Test for overall effect: Z = 0.59 (P = 0.56)						
**1.43.4 5-year postoperative mortality and morbidities(%)**						
Zhang Y 2018d	7	100	1	102	100.0%	7.60 [0.92, 62.96]
**Subtotal (95% CI)**		**100**		**102**	**100.0%**	**7.60 [0.92, 62.96]**
Total events	7		1			
Heterogeneity: Not applicable						
Test for overall effect: Z = 1.88 (P = 0.06)						
**1.43.5 7-year postoperative mortality and morbidities(%)**						
Zhang Y 2018c	0	108	2	80	35.6%	0.14 [0.01, 3.06]
Zhang Y 2018d	18	100	9	102	64.4%	2.27 [0.97, 5.33]
**Subtotal (95% CI)**		**208**		**182**	**100.0%**	**0.85 [0.06, 11.59]**
Total events	18		11			
Heterogeneity: Tau² = 2.56; Chi² = 2.96, df = 1 (P = 0.09); I² = 66%						
Test for overall effect: Z = 0.12 (P = 0.90)						
**1.43.6 8-year postoperative mortality and morbidities(%)**						
Zhang Y 2019b	7	161	7	225	100.0%	1.42 [0.49, 4.12]
**Subtotal (95% CI)**		**161**		**225**	**100.0%**	**1.42 [0.49, 4.12]**
Total events	7		7			
Heterogeneity: Not applicable						
Test for overall effect: Z = 0.64 (P = 0.52)						

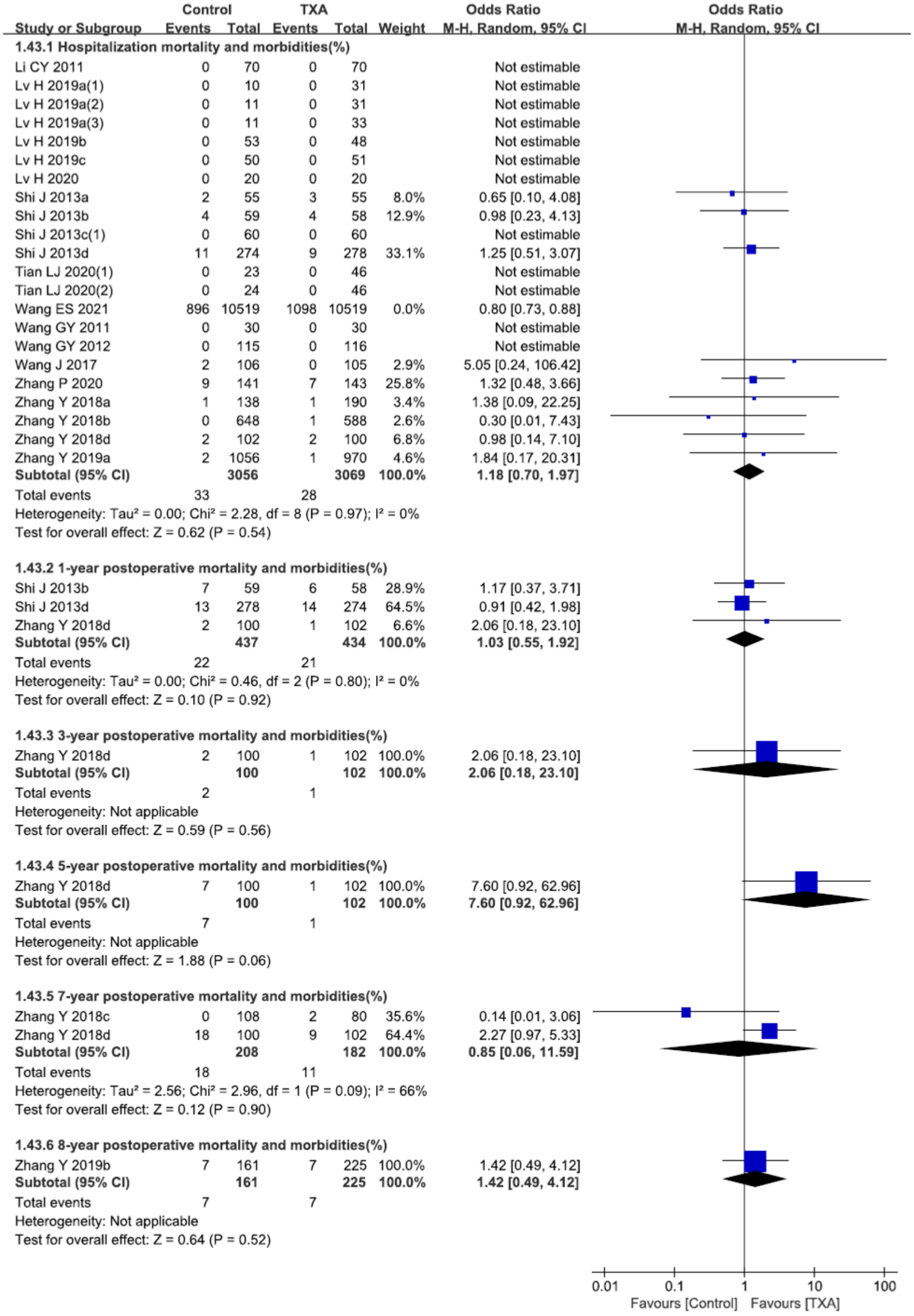

Supplement: Supplementary file 6 [file medi-102-e33819-s006.pdf]
