# Supplementary material for: The efficacy and safety of intravenous administration of tranexamic acid in patients undergoing cardiac surgery: Evidence from a single cardiovascular center
Source: Medicine (Baltimore). 2023 May 17;102(20):e33819. doi: 10.1097/MD.0000000000033819 (PMC10194539; doi:10.1097/MD.0000000000033819)
Supplement: Supplementary file 7 [file medi-102-e33819-s007.pdf]

Supplement Table 4. The effects of TXA on intraoperative heparinization, reversal and other coagulation variables

Outcomes	Studies(n)	Patients(n)	Heterogeneity *P* value	I^2	WMD	OR	95%CI	Overall effect *P* value
**Intra-op heparin dose**(u)								
①*Adults*								
TXA *vs*. CTRL	5	973	0.31	16%	-2.99	-	-6.25, 0.27	0.07
②*Pediatrics*								
TXA vs. CTRL	0							
**Intra-op protamine dose**(mg)								
①*Adults*								
TXA *vs*. CTRL	6	1,257	0.95	0%	6.50	-	-3.76, 16.77	0.21
②*Pediatrics*								
TXA vs. CTRL	0							
**Protamine/heparin dose ratio**(ratio)								
①*Adults*								
TXA *vs*. CTRL	6	1,257	0.68	0%	0.02	-	-0.00, 0.05	0.09
②*Pediatrics*								
TXA vs. CTRL	0							
**ACT at EOS**(sec)								
①*Adults*								
TXA *vs*. CTRL	1	231	NA	NA	1.00	-	-2.23, 4.23	0.54
②*Pediatrics*								
TXA vs. CTRL	0							
**PT on POD-1**(sec)								
①*Adults*								
TXA *vs*. CTRL	2	291	0.10	64%	0.08	-	-0.68, 0.85	0.83

TXA(LD) *vs.* TXA(MD)	1	158	NA	NA	0.00	-	-0.39, 0.39	1.00
TXA(LD) *vs.* TXA(HD)	1	159	NA	NA	0.10	-	-0.30, 0.50	0.63
TXA(MD) *vs.* TXA(HD)	1	157	NA	NA	0.10	-	-0.29, 0.49	0.62
②*Pediatrics*								
TXA vs. CTRL	0							
**PT-INR on POD-1**								
①*Adults*								
TXA *vs.* CTRL	2	291	0.24	29%	0.08	-	0.03, 0.14	0.001*
TXA(LD) *vs.* TXA(MD)	1	158	NA	NA	0.00		-0.03, 0.03	1.00
TXA(LD) *vs.* TXA(HD)	1	159	NA	NA	0.10		0.07, 0.13	<0.00001*
TXA(MD) *vs.* TXA(HD)	1	157	NA	NA	0.10		0.07, 0.13	<0.00001*
②*Pediatrics*								
TXA vs. CTRL	0							
**D-dimer on POD-1**(mg/L)								
①*Adults*								
TXA *vs.* CTRL	1	60	NA	NA	-0.58	-	-0.77, -0.39	<0.00001*
TXA(LD) *vs.* TXA(MD)	1	158	NA	NA	0.00	-	-0.11, 0.11	1.00
TXA(LD) *vs.* TXA(HD)	1	159	NA	NA	0.10	-	0.01, 0.19	0.03*
TXA(MD) *vs.* TXA(HD)	1	157	NA	NA	0.10	-	0. 03，0. 17	0.005*
②*Pediatrics*								
TXA vs. CTRL	0							
**Platelet counts on POD-1**(×10^9/L)								
①*Adults*								
TXA vs. CTRL	1	231	NA	NA	2.00	-	-8.96, 12.96	0.72
TXA(LD) *vs.* TXA(MD)	2	258	0.83	0%	4.21		-4.90, 13.33	0.36

TXA(LD) *vs.* TXA(HD)	2	258	0.83	0%	3.26		-5.52, 12.04	0.47
TXA(MD) *vs.* TXA(HD)	2	258	1.00	0%	-1.00		-9.50, 7.50	0.82
②*Pediatrics*								
TXA vs. CTRL	1	30	NA	NA	13.00	-	-14.20, 40.20	0.35
**GMP-140+ platelet percentage at EOS**(%)								
①*Adults*								
TXA vs. CTRL	0							
②*Pediatrics*								
TXA vs. CTRL	1	30	NA	NA	-1.16	-	-4.04, 1.72	0.43
**TXB2 on POD-1**(ng/L)								
①*Adults*								
TXA vs. CTRL	1	101	NA	NA	2.70	-	-18.71, 24.11	0.80
②*Pediatrics*								
TXA vs. CTRL	0							

ACT=activated clotting time, CI=confidence interval, CTRL=control, EOS=end of surgery, GMP-140=guanosine membrane protein-140, INR=international normalized ratio, OR=odds ratio, POD=post-operative day, Post-op=post-operative, PT=prothrombin time, TXA=tranexamic acid, TXB2=thromboxane B2, WMD=weighted mean difference.
