# Supplementary material for: The efficacy and safety of intravenous administration of tranexamic acid in patients undergoing cardiac surgery: Evidence from a single cardiovascular center
Source: Medicine (Baltimore). 2023 May 17;102(20):e33819. doi: 10.1097/MD.0000000000033819 (PMC10194539; doi:10.1097/MD.0000000000033819)
Supplement: Supplementary file 8 [file medi-102-e33819-s008.pdf]

Supplement Table 5. The effects of TXA on post-operative inflammation and organ injury biomarkers

Outcomes	Studies(n)	Patients(n)	Heterogeneity *P* value	I^2	WMD	OR	95%CI	Overall effect *P* value
**WBC counts**($\times 10^9$/L)								
①*Adults*								
TXA *vs.* CTRL	1	139	0.08	67%	0.14	-	-2.21, 2.49	0.91
TXA(LD) *vs.* TXA(MD)	1	100	NA	NA	0.10	-	-1.29, 1.49	0.89
TXA(LD) *vs.* TXA(HD)	1	99	NA	NA	0.20	-	-1.24, 1.64	0.79
TXA(MD) *vs.* TXA(HD)	1	101	NA	NA	0.10	-	-1.34, 1.54	0.89
②*Pediatrics*								
TXA vs. CTRL	0							
**IL-6**(pg/ml)								
①*Adults*								
TXA *vs.* CTRL	1	60	NA	NA	-26.00	-	-35.37, -16.63	<0.00001*
②*Pediatrics*								
TXA vs. CTRL	0							
**PMNE**(ng/ml)								
①*Adults*								
TXA *vs.* CTRL	1	40	NA	NA	-226.90		-353.03, -100.77	0.00041*
②*Pediatrics*								
TXA vs. CTRL	0							
**Fibronectin**(μg/ml)								
①*Adults*								
TXA *vs.* CTRL	1	60	0.90	0%	188.85		100.41, 277.29	<0.0001*
TXA(LD) *vs.* TXA(MD)	1	40	NA	NA	-11.00		-136.56, 114.56	0.86
TXA(LD) *vs.* TXA(HD)	0							

TXA(MD) *vs.* TXA(HD)	0							
②*Pediatrics*								
TXA vs. CTRL	0							
**cTnI**(mg/L)								
①*Adults*								
TXA *vs.* CTRL	1	140	NA	NA	-0.32	-	-0.78, 0.14	0.17
②*Pediatrics*								
TXA vs. CTRL	0							
**CK-MB**(U/ml)								
①*Adults*								
TXA *vs.* CTRL	1	140	NA	NA	-13.00	-	-18.64, -7.36	<0.00001*
②*Pediatrics*								
TXA vs. CTRL	0							
**Cr**(μ mol/L)								
①*Adults*								
TXA *vs.* CTRL	1	231	NA	NA	-6.60	-	-13.84, 0.64	0.07
TXA(LD) *vs.* TXA(MD)	2	258	0.49	0%	3.29	-	-3.77, 10.35	0.36
TXA(LD) *vs.* TXA(HD)	2	258	0.18	0%	-0.84	-	-7.95, 6.27	0.82
TXA(MD) *vs.* TXA(HD)	2	258	0.63	0%	-3.60	-	-10.80, 3.60	0.33
②*Pediatrics*								
TXA vs. CTRL	0							

CI=confidence interval, CK=creatine kinase, Cr=creatinine, cTnI=cardiac troponin I, CTRL=control, HD=high dose, IL-6=interleukin-6, LD=low dose, MD=medium dose, NA=not applicable, OR=odds ratio, PMNE=polymorphonuclear leukocyte elastase, TXA=tranexamic acid, WBC=white blood cell, WMD=weighted mean difference.
