# Supplementary material for: The efficacy and safety of intravenous administration of tranexamic acid in patients undergoing cardiac surgery: Evidence from a single cardiovascular center
Source: Medicine (Baltimore). 2023 May 17;102(20):e33819. doi: 10.1097/MD.0000000000033819 (PMC10194539; doi:10.1097/MD.0000000000033819)
Supplement: Supplementary file 2 [file medi-102-e33819-s002.pdf]

**Supplement Table 1.** Quality assessment of included cohort studies using Newcastle-Ottawa Scale

| Study | Newcastle-Ottawa Scale | | | | | | | | |
|---|---|---|---|---|---|---|---|---|---|
| | Selection | | | | Comparability | Outcome | | | Total |
| | Representativeness of Cohort | Selection of Non-exposed Cohort | Ascertainment of Exposure | Demonstration That Outcome of Interest Was Not Present at Start of Study | Comparability of Cohorts on Basis of Design or Analysis Time to Follow-Up | Assessment of Outcome | Follow-Up Long Enough for Outcomes to Occur | Adequacy of Follow-Up Cohorts | |
| Wang ES 2020[33] | 1 | 1 | 1 | 1 | 2 | 1 | 1 | 1 | 9 |
| Liu Y 2021[34] | 1 | 1 | 1 | 1 | 1 | 1 | 0 | 1 | 7 |
| Zhang Y 2019[35] | 1 | 1 | 1 | 1 | 2 | 1 | 1 | 1 | 9 |
| Zhang Y 2018a[36] | 1 | 1 | 1 | 1 | 1 | 1 | 1 | 1 | 8 |
| Zhang Y 2018b[37] | 1 | 1 | 1 | 1 | 1 | 1 | 1 | 1 | 8 |
